# The GPVI – Fc Fusion Protein Revacept Reduces Thrombus Formation and Improves Vascular Dysfunction in Atherosclerosis without Any Impact on Bleeding Times

**DOI:** 10.1371/journal.pone.0071193

**Published:** 2013-08-12

**Authors:** Martin Ungerer, Zhongmin Li, Christine Baumgartner, Silvia Goebel, Jasmin Vogelmann, Hans-Peter Holthoff, Meinrad Gawaz, Götz Münch

**Affiliations:** 1 advanceCOR GmbH (formerly, Procorde GmbH), Martinsried, Germany; 2 Medizinische Klinik III, Kardiologie und Kreislauferkrankungen, Eberhard Karls Universität Tübingen, Tübingen, Germany; William Harvey Research Institute, Barts and The London School of Medicine and Dentistry, Queen Mary University of London, United Kingdom

## Abstract

**Aims:**

Glycoprotein VI (GPVI) is a key platelet receptor which mediates plaque-induced platelet activation and consecutive atherothrombosis, but GPVI is also involved in platelet-mediated atheroprogression. Therefore, interference in GPVI-mediated platelet activation has the potential to combine short-term and long-term beneficial effects, specificity and safety especially regarding bleeding complications.

**Methods and Results:**

We investigated the effects of the soluble dimeric GPVI receptor fusion protein, Revacept, an antagonist of collagen-mediated platelet activation, in an animal model of atherosclerosis: twenty week old rabbits, which had been fed on a cholesterol-rich diet for 8 weeks, received Revacept (8 mg/kg) or control twice weekly for 4 weeks. Pharmacokinetics indicated a slight accumulation of the drug in the serum after repeated dosing of Revacept for 3 weeks. A significant improvement of endothelial dysfunction after 0.06 and 0.6 µg/min acetylcholine and a significant decrease of vessel wall thickening were found after Revacept treatment. Accordingly, aortic vessel weight was reduced, and plaque sizes, macrophage and T-cell invasion tended to be reduced in histological evaluations. Bleeding time was determined after tail clipping in mice. Revacept alone or in combination with widely used anti-platelet drugs revealed a high safety margin with no prolongation of bleeding times.

**Conclusion:**

Repeated doses of Revacept led to a significant improvement of endothelial dysfunction and vascular morphology in atherosclerotic rabbits. Furthermore, no influence of Revacept on bleeding time alone or in combinations with various anti-platelet drugs was found in mice. Thus, the inhibition of collagen-mediated platelet interaction with the atherosclerotic endothelium by Revacept exerts beneficial effects on morphology and vascular function in vivo and seems to have a wide therapeutic window without influencing the bleeding time.

## Introduction

Rupture of atherosclerotic plaques leads to adhesion of circulating platelets to exposed sub-endothelial matrix proteins which trigger subsequent platelet activation and aggregation. Among the macromolecular components of the sub-endothelial layer, fibrillar collagen is considered to be the most thrombogenic constituent, as it acts as a strong activator of platelets and supports platelet adhesion both in vitro and in vivo [1], [2].

Among the multitude of different platelet receptors, GPVI – a ∼68 kilo Dalton Type I trans-membrane glycoprotein receptor that interacts with collagen to trigger platelet activation and aggregation – plays a key role in plaque-mediated thrombus formation [3]. Moreover, GPVI is also involved in chronic platelet interaction with the activated atherosclerotic endothelium and in platelet-mediated progression of atherosclerosis [4], [5]. Published data suggest that platelets might significantly contribute to the inflammatory process that promotes atherosclerotic lesion formation [6], [7].

Inhibition of the GPVI pathway by anti-GPVI antibodies ameliorates atherosclerosis in ApoE −/− ablated mice [8]. Another possibility to interfere in the GPVI pathway is the soluble GPVI receptor Revacept [1]. Revacept is a dimeric recombinant fusion protein consisting of the Fc part of a human immunoglobulin G (IgG) together with the hinge region and the functional GPVI domain at the N-terminus (GPVI-Fc) [1]. This novel anti-platelet tool has been proven beneficial in various animal models of acute vascular injury [1], [9]. Moreover, the safety and efficacy was demonstrated in a controlled phase I study in man [10]. Human GPVI-Fc/Revacept is thought to act by blocking the binding sites of platelets at collagen, fibronectin [8], [11] and possibly other vascular ligands, such as von Willebrand factor, and therefore might interfere with platelets not only in acute plaque rupture but also during chronic interaction with the activated but intact atherosclerotic endothelium.

In this study, we therefore aimed to clarify the role of GPVI on platelet-mediated plaque progression. Moreover, we investigated the role of the platelet activation inhibitor Revacept as a novel tool to interfere in platelet-triggered vessel damage by studying the effects on the functional and morphological consequences of atherosclerosis. Cholesterol-fed rabbits are a suitable model for the in vivo investigation of atherosclerosis [8]. Finally, we also investigated the safety of this novel drug by testing drug interaction with other established anti-platelet drugs on bleeding time.

## Materials and Methods

All animal experiments were approved by the local animal welfare authority and Ehtics committee at the Regierung von Oberbayern (Government of Upper Bavaria) in Munich, Germany (reference number 209.1/211-2531.-37/04) and carried out in accordance to the European Commission guidelines.

### Animals

New Zealand White (NZW) rabbits (aged 20 weeks, from Asam, Kissing, Germany) were used according to international and national guidelines for animal health. A permission to carry out animal studies was granted from the local animal safety supervising authority (study numbers 209.1/211-2531.-37/04). Standard rabbit chow and water were freely available. For the induction of atherosclerosis, the animals were fed a cholesterol-rich diet (1% cholesterol 5% corn oil; Altromin GmbH, Lage, Germany) from the age of 20 weeks until the end of experiment (when the animals were aged 28 weeks).

Nine week old male black six mice (C57Bl/6J) were obtained from Charles River, Sulzfeld, Germany, and used in bleeding experiments.

### Carotid Artery Endothelial Denudation

**Figure 1:**
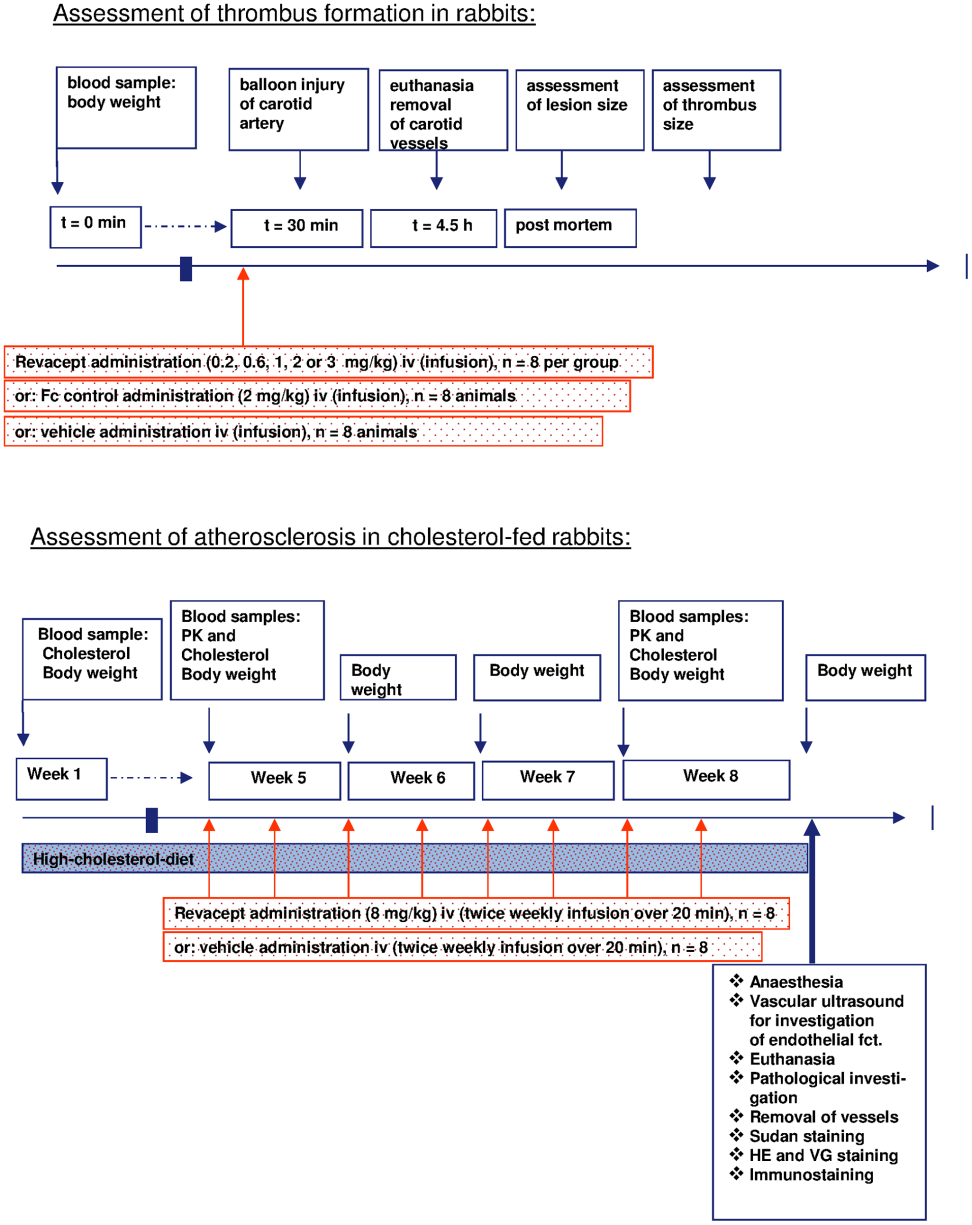
Study protocols of thrombus size detection after carotis artery lesion (upper panel), and of atherosclerosis in cholesterol-fed rabbits after eight weeks of drug administration (lower panel).

Under general anaesthesia (Propofol 1%, Fresenius Kabi, Bad Homburg, Germany), the left femoral artery was prepared, an embolectomy catheter (3F, Edwards Lifesciences) was introduced in the right carotid artery and endothelial denudation was carried out between the second and sixth cervical vertebrae by gently moving the inflated (with 0.4 ml of air) balloon catheter backwards and forwards twice. 30 min before damage to the right carotid artery was induced, the rabbits (n = 69) were given Revacept (batch 260805), Fc control (batch CP31) or PBS by continuous iv infusion via the ear vein of 5 ml over a period of 3 min. The doses used were: 0.2 mg/kg; 0.6 mg/kg; 1 mg/kg; 2 mg/kg; and 3 mg/kg PR-15; and 2 mg/kg Fc control. Please see Figure 1 (upper panel) for an overview on the protocol.

### Evaluation of the Denudation after Balloon Injury

For investigation of the position and extent of the endothelial lesion produced by the balloon, rabbits were given Evans Blue (2%, 1 ml/kg) iv after endothelial denudation, as described previously (e.g., Ohtsuki K, Hayase M, Akashi K et al., Detection of MCP-1 receptor expression in experimental atherosclerotic lesions. Circulation 2001; 104: 203–208). After euthanasia with pentobarbital iv (60 mg/kg, Narcoren®, Merial GmbH, Germany), paraformaldehyde was administered iv (2% in PBS, for 10 minutes, 100 mm Hg pressure) for tissue fixation. The right and left common carotid arteries were removed, cleaned of adventitia and connective tissue, and segments of the arteries (starting 0.6 cm from the distal end of the brachiocephalic trunk and ending at the carotid bifurcation 6 cm distal from the starting position) were opened longitudinally with the endothelium facing up and pinned flat on a white wax plate.

A digital picture of each prepared vessel was taken with a Canon EOS 300 camera, and the lesion area was calculated from the digital picture using computer-assisted image analysis software. This analysis quantified the blue (damaged) area of the lesion in pixels and the total area of the complete vessel segment in pixels (Scion image; Scion Corporation, Frederick, MD, USA). The total area (expressed as pixels) varied due to different resolutions of the digital pictures. Therefore, the lesion area was calculated as percent of the total area. In addition, sections of tissue from both the right and left carotid arteries were cut, fixed, processed and stained with haematoxylin and eosin and Elastic van Gieson staining to evaluate the degree of denudation and depth of the lesion.

### Quantification of Thrombus Formation

Four hours after local or systemic drug administration, the left and right common carotid arteries were fixed by iv perfusion with paraformaldehyde (2% in PBS, for 10 min, via a catheter through the abdominal aorta, 100 mm Hg pressure) and removed. They were cleaned of adventitia and connective tissue, and segments of the arteries were opened longitudinally with the endothelium facing up.

After mounting the segments on slides, the insides of the vessels were examined macroscopically. Thrombi were easily visible by their red colour against the white background of the vessel wall. A digital picture of the prepared vessel was then taken with a Canon EOS 300 camera and the area covered by thrombi was quantified from the digital picture using computer-assisted image analysis software (Scion image; Scion Corporation, Frederick, MD, USA), which detected the red thrombus area in pixels and the total area of the complete vessel segment in pixels. The total area was thus the same in all the arteries analysed. Again, the total area varied due to the different resolutions of the digital pictures. Therefore, the area covered by thrombi was calculated as a proportion of the total area.

### Atherosclerosis

16 rabbits were randomly assigned to receive either Revacept or vehicle control. Blood samples were taken to investigate baseline and high-cholesterol diet serum cholesterol levels. Eight rabbits were infused with Revacept (8 mg/kg in 3.33 ml/kg formulation buffer) intravenously (iv) via the ear vein twice weekly for four weeks. This dose had been chosen on the basis on prior ex vivo investigation of foam cells. Eight animals were given formulation buffer (PBS pH 7.4, 4% mannitol, 1% sucrose), and served as controls (control group). The pharmacokinetic profiling of Revacept was carried out on day 1 and 22 of the dosing period. Please see Figure 1 (lower panel) for an overview on the protocol. The general condition of the animals was checked daily and rabbits were weighed weekly before and twice weekly after start of drug infusions, respectively.

When the animals had reached an age of 28 weeks, endothelial function was investigated in vivo by vascular ultrasound under general anaesthesia (Propofol 1%, Fresenius Kabi, Bad Homburg, Germany). To this end, acetylcholine-induced vasoreactivity, and vessel wall thickness were determined. Animals were sacrificed and the heart, the thoracic and abdominal aorta and both common carotid arteries were removed and stored at −80°C until required for histological examinations. The investigators were blinded during the complete in-life part of the study as well as during the histological evaluation post mortem.

### Determination of Serum Cholesterol Levels

Serum cholesterol was determined before and in week 5 and 8 of cholesterol-rich diet with a commercial photometric method (Synlab vet, Augsburg, Germany).

### Analytical Methods for Assessment of PK Profiles

Revacept concentrations were determined according to the manufacturer’s instructions using a commercial ELISA Kit (Immuno-Tek, ZMC Cat. No. 0801182).

### Calculation and Interpretation of the PK Results

Revacept pharmacokinetics were calculated with a non-compartmental analysis (NCA) using PCModfit (version 3.00) for Windows® running with Excel 2003 (version 11.8). Half-life, terminal elimination rate constant (λz) and area under the concentration-time curve to the last time point (AUC0-t) and area to infinity (AUC0-∞) was estimated using AUC0-t were calculated.

### Investigation of Endothelial Function in Atherosclerosis

At an animal age of 24 weeks and 28 weeks, the endothelial function was investigated under general anaesthesia (Propofol 1%, Fresenius Kabi, Bad Homburg, Germany) iv. Monitoring of mean heart rate (HR), end-tidal CO2 (EtCO2) and arterial oxygen saturation (SpO2) was carried out using a Datex device (Datex Ohmeda S/5, Type F-CM1.00, Helsinki, Finland, pressure transducer: Hellige Type 4-327-I). Vascular ultrasound was carried out over the right ACC using a 10 MHz linear transducer (FLA 10 MHz 1A, GE Vingmed, Horten, Norway) in conjunction with a Vingmed ultrasound system (A/S System FIVE/REM, GE, Horten, Norway). Endothelial function was investigated by continuous infusion of increasing concentrations of acetylcholine (Acetylcholine chloride, Sigma-Aldrich Chemie GmbH, Steinheim, Germany). A group of six healthy New Zealand White rabbits which had been kept at regular chow, and were also aged 28 weeks, was additionally compared to the cholesterol-fed rabbits. Vessel luminal diameter at maximum vessel dilation and vessel wall thickness were determined before and after increasing doses of acetylcholine (0.006 µg/min, 0.06 µg/min and 0.6 µg/min).

### Macroscopic Assessment of Atherosclerosis

After sacrifice of the animals, the aortae (including the brachiocephalic trunk) and the left and right common carotid arteries were carefully removed and frozen in liquid nitrogen. The vessels were macroscopically prepared for “en face” evaluation of plaque extension. After overnight post-fixation in 4% formalin, the arteries were stained with Sudan red. The endothelium facing up photographs with a Canon EOS 300 camera were taken. Digital pictures were analysed (Scion image; Scion Corporation, Frederick, MD, USA), and relative plaque area was calculated in percent of the total vessel area.

### Histological Evaluation of Intima and Media Area in Brachiocephalic Trunk

Vessels were freeze-cut in sequential 6-µm sections and standard Haematoxylin and eosin (HE) and van Gieson elastic (VG) staining were performed (HT25, Sigma Diagnostics, Louis, UpSA).

Digital images were obtained and the intima and media areas were assessed by computer-assisted image analysis (as described above). For immunohistochemical analysis, the sections were post-fixed with acetone and counterstained with Harris haematoxylin. As primary antibodies, mouse monoclonal anti-rabbit RAM 11 antibody (DAKO, Hamburg, Germany, used at a dilution of 1∶50) were used for macrophages, mouse monoclonal anti-rabbit CD 43 (Serotec, used at 0.33 mg/ml) for T-cells. Biotinylated anti-mouse (DAKO, Hamburg, Germany) antibodies were used as secondary antibodies, at a titer of 1∶300, as recommended by the manufacturer. Antibody binding was visualised by incubation with the substrate 3′3′-diaminobenzidine (DAB).

### Quantification of the Cell Density by Immunohistochemistry

Serial sections of 5 µm were cut every 75 µm along the brachiocephalic artery and every 80 µm along the 230-µm segment of the aortic roots. The first tissue section of every segment was stained with hematoxylin/eosin. Adjacent sections were stained with oil red O, Sirius red, Movat’s pentachrome, and Elastic van Gieson and immunostained for the visualization. Macrophage and T-cell numbers were determined by counting blue nuclei in positively stained cells within lesions. The investigator was blinded during this histological evaluation.The relative content of the immune cells was then calculated by forming the ratio of the number of specific cells and the plaque area, evaluated in adjacent van Gieson elastic stained sections. The analysis of lesions was performed in a blinded fashion.

### Bleeding Time Measurement in Rabbits

For investigation of primary bleeding time in atherosclerotic rabbits, animals were treated with propofol (1%, 5–8 mg/kg iv with continuing low-dose administration), until deep anaesthesia was reached. Then, a standardized incision with a length of 5 mm and a depth of 1 mm was placed in the right ears of rabbits with a number 11 surgical blade. The ear was then immersed in a beaker containing saline warmed around 38.5°C, and duration of bleeding was measured. Revacept (2 mg/kg body weight) or vehicle were given 30 minutes prior to measurement by iv bolus injection via a needle which had been placed in a contralateral ear vein.

### Bleeding Time Measurement in Mice and the Influence of Drug Interactions on the Safety of Revacept

For investigation of the bleeding time, the tail transsection method was used which is a well-established technique in mice. Except for clopidogrel, acetyl salicylic acid in combination with dipyridamol, all drugs and saline were administered intravenously to conscious animals (restrained in an injection chamber) in a volume of 100 µl. The tails were transsected at the time of the anticipated approximate C_max_ or maximal anti-coagulative/anti-platelet effect in mice: Revacept 30 min, aspirin 60 min and heparin 10 min. Clopidogrel, dissolved in sodium chloride with a volume of 200 µl, was given orally by gavage under short-term anaesthesia with isoflurane 120 minutes before tail transsection.

For determination of bleeding time, the mice were anaesthetised with medetomidine (Domitor® 500 µg/kg), midazolam (Dormicum® 5 mg/kg) and fentanyl (Fentanyl B. Braun 50 µg/kg) intraperitoneally 10 min prior to tail transsection. The distal 2 mm of the animalś tail was sectioned with a scalpel blade and the tail immersed in warm buffer solution (PBS). Bleeding time was recorded immediately following transection and was continued until blood ceased to flow from the tip of the tail without restarting within 30 seconds.

The effect of Revacept (2 mg/kg BW) on bleeding time was compared to animals receiving various doses of clopidogrel, acetyl salicylic acid (Aspirin, ASA) dipyridamol and heparin, and combinations of those. The doses of the comparator drugs corresponded to the maximum doses approved for therapeutic use in humans, and were calculated for use in mice on a per body weight basis. The dose of ASA (14 mg/kg BW) is comparable to that which is given to humans in a 70–75 kg patient. Clopidogrel 1 mg/kg BW corresponds to the recommended human dose of 75 mg daily, while 5 and 10 mg/kg BW correspond to the loading doses of clopidogrel (300 and 600 mg).

Additionally, the daily therapeutic doses of Aggrenox (ASA 50 in combination with dipyridamole 400 mg) in combination with acetyl salicylic acid (100 mg) were tested after p.o. applications in mice. The body weight doses for acetyl salicylic acid in combination with dipyridamole of 2 mg/kg BW and of 5.3 mg/kg BW were calculated on the basis of a 75 kg patient.

### Statistical Analysis

The results were tested for Gaussian distribution using the Kolmogorow-Smirnov test. If this test showed normal distribution of the values, an ANOVA followed by post-hoc Bonferronís analysis was used to compare groups. Where appropriate, a t-test for independent samples was used. P-values <0.05 were considered statistically significant.

## Results

### Endothelial Lesion after Balloon Injury

Reproducible thrombus formation developed in all experiments at the sites of Fogarty balloon catheter-induced injury of the right carotid artery of rabbits with little variability between experiments. The areas of endothelial injury were confirmed by Evans Blue staining. By semi-quantitative computer-assisted image analysis, 76±6% of the vessel area of the right common carotid artery that was digitally analysed showed a lesion of the endothelium. Staining with haematoxylin and eosin and Elastic van Gieson confirmed that the internal tunica elastica was intact and that the lesion was limited to the endothelium. Bleeding and coagulation within the media or swelling of the vessel wall that could have affected blood flow could be excluded.

### Effect of Revacept on Thrombus Formation after Balloon Injury

**Figure 2:**
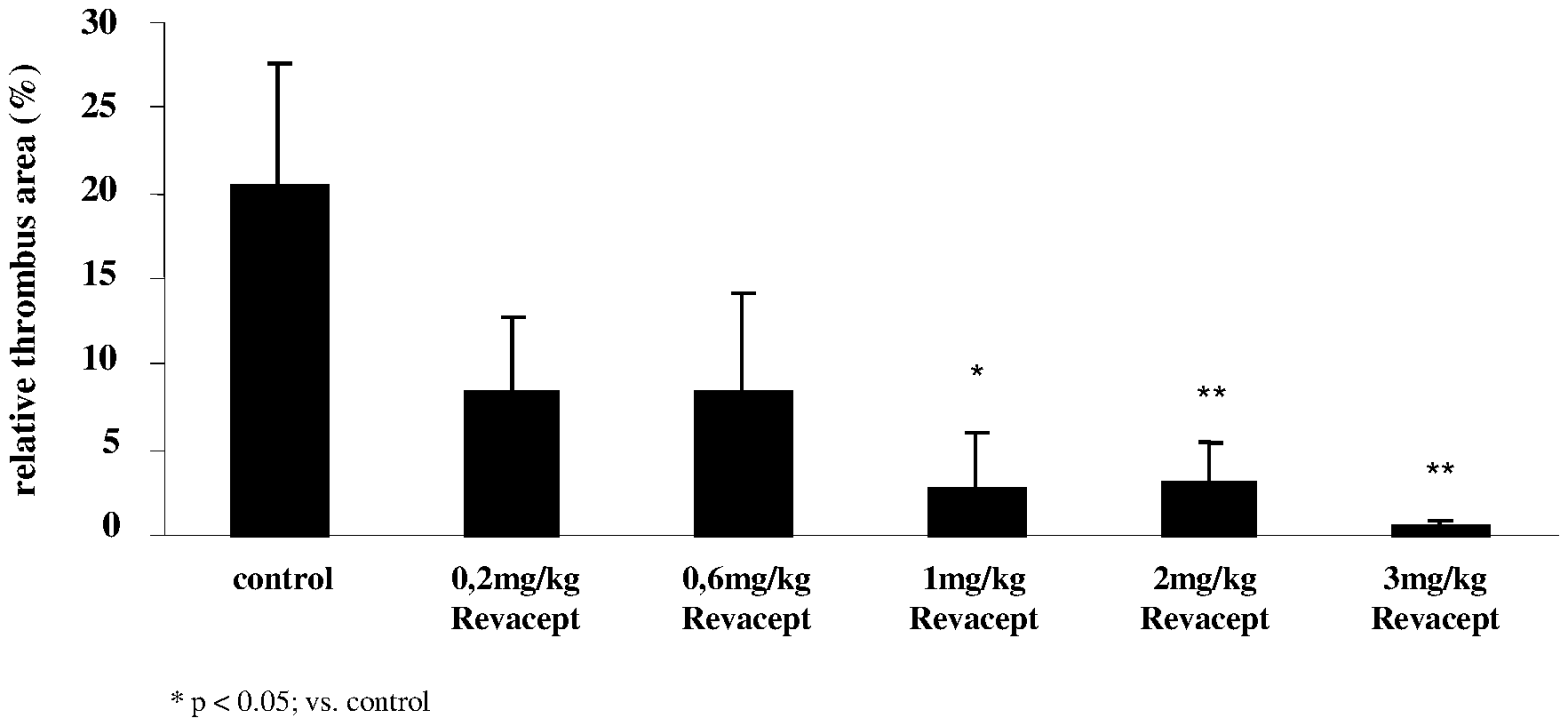
Effect of systemic delivery of increasing doses of Revacept in rabbits. Thrombus formation was induced by balloon vascular injury. The thrombus size was evaluated histologically post mortem and is expressed as % of the total vascular lesion area. The mean ± SD of n = 8 experiments per group are shown. * indicates significant difference of p<0.05, and ** of p<0.01, versus controls (as determined by ANOVA).

**Figure 3:**
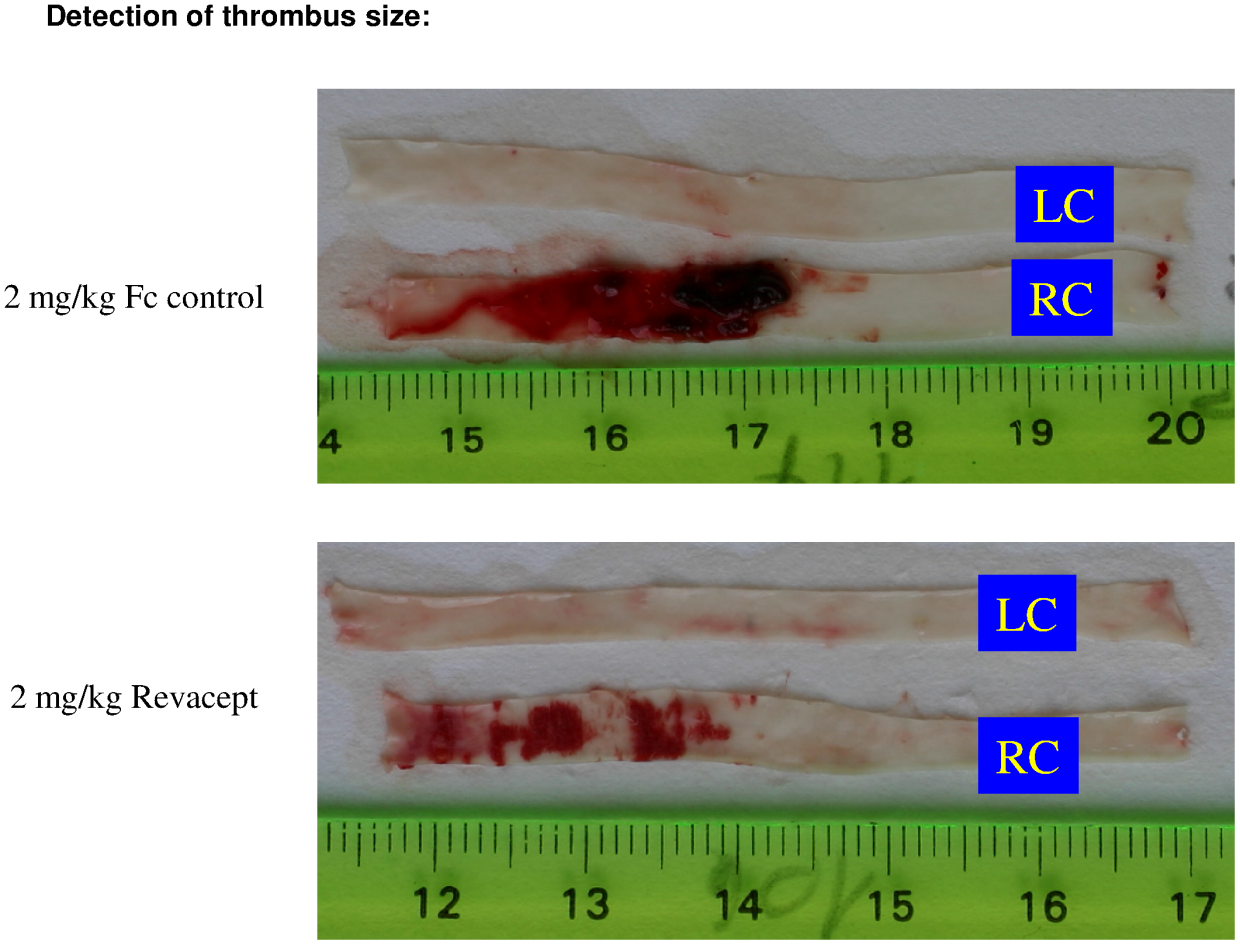
Representative images of thrombi in carotid artery preparations (en face). LC: left carotid artery; RC: right carotid artery.

Single administration of Revacept 30 min prior to denudation at doses of 1 mg/kg, 2 mg/kg and 3 mg/kg iv produced a statistically significant reduction in thrombus formation compared to the group treated with the Fc control/PBS (see Figure 2). Representative photographic images are shown in Figure 3. The lower doses of Revacept also reduced thrombus formation. However, these differences did not reach statistical significance. The dose range used in this study was already used in several previous studies, and shown to be effective [1].

### Pharmacokinetic Profile of Revacept

**Figure 4:**
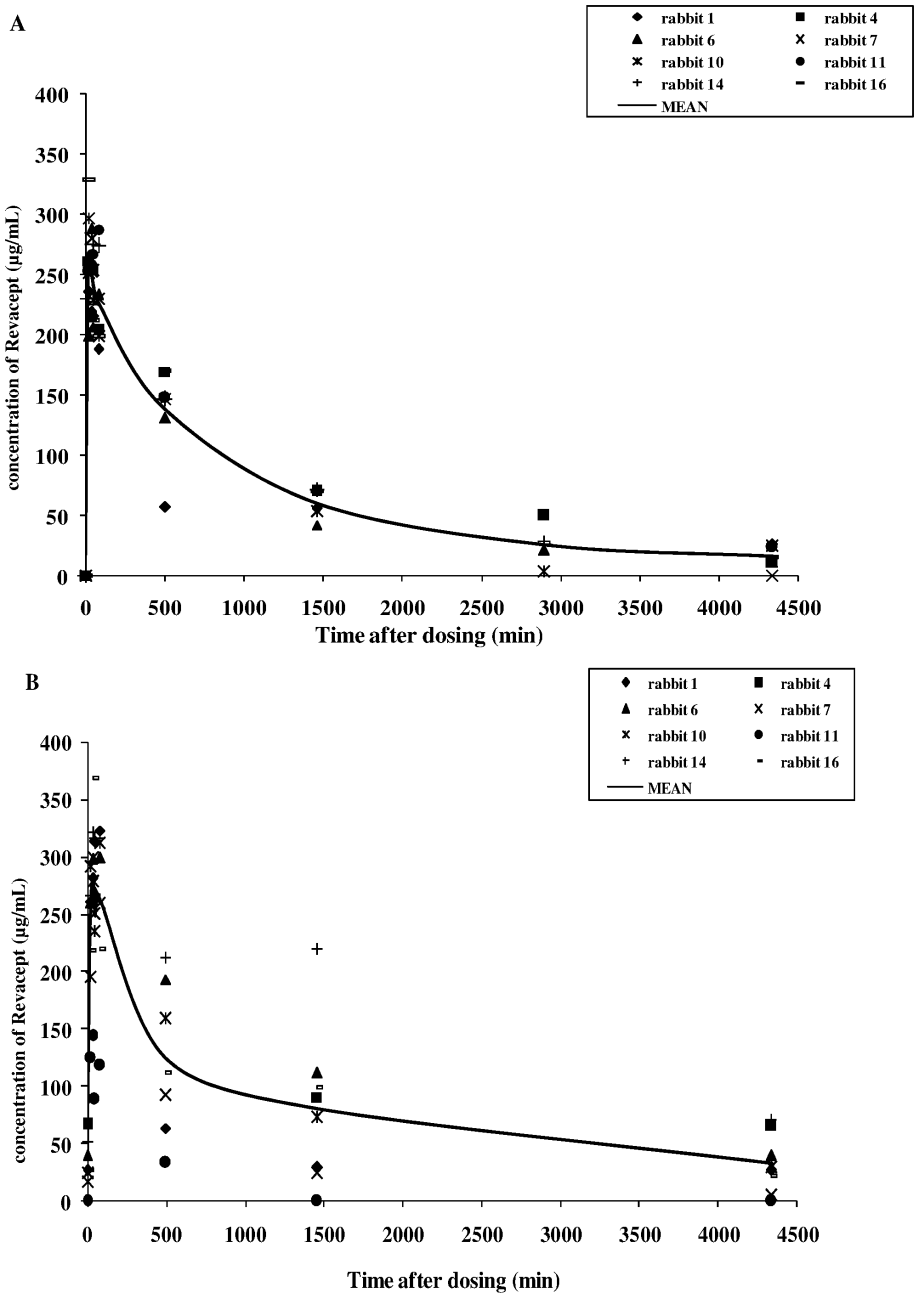
Pharmacokinetic profile of Revacept in rabbits (A) on the first day of dosing (after 4 weeks of high cholesterol feeding), (B) after the last dosing of the twice weekly dosing period four weeks later. The graphs show single animal and mean values of the Revacept group.

After repeated IV administration of Revacept (8 mg/kg) in another group of rabbits, the mean C_max_ values were 283.1 µg/mL on the first day and 320.4 µg/mL on the 22nd day, respectively, indicating only a slight accumulation of the drug in the serum after repeated dosing of Revacept (please see Figures 4 A and B). The mean t_1/2_ of Revacept was 25.6 h on the first day of dosing and 31.9 h on the 22nd day after repeated doses. Dosing of 3 or 2 mg/kg BW Revacept had previously resulted in similar curve shapes, and proportionately lower C_max_ values. The variability of the PK assay was low with a CV between 11.2 to 27.8% at consecutive time points. No drug was detected in the serum samples of the control animal.

### Serum Cholesterol Levels and Body Weight

**Figure 5:**
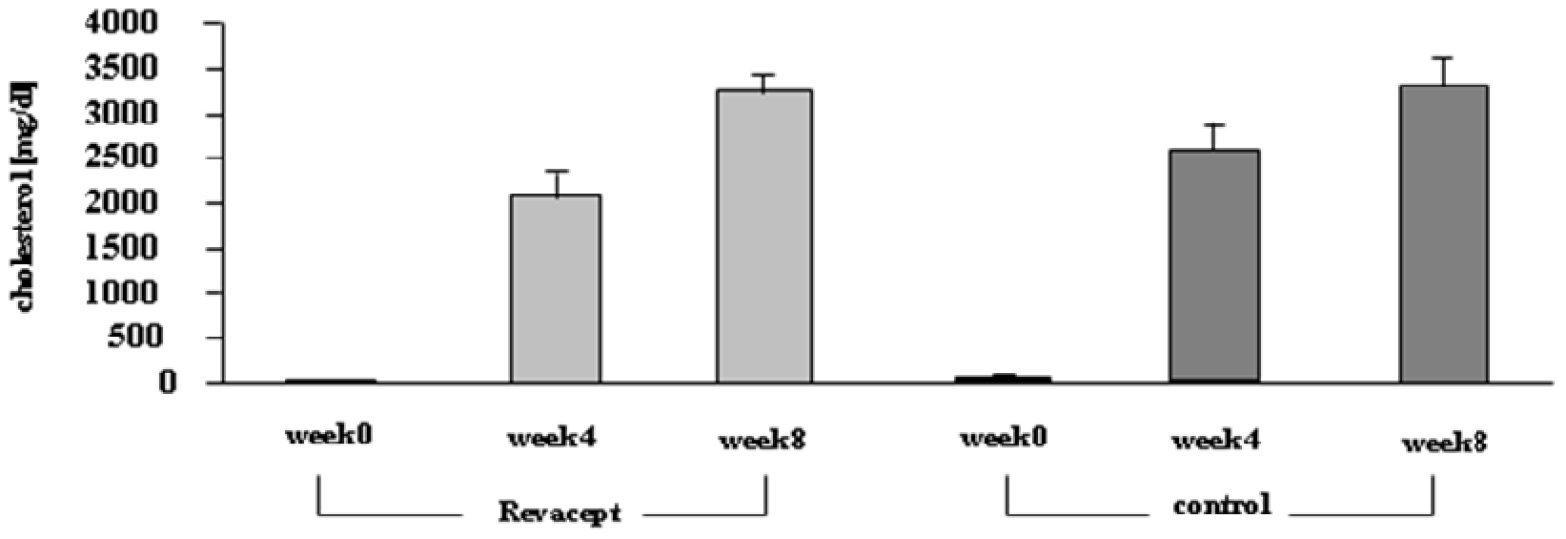
Serum cholesterol levels of the Revacept group and the control group before, 4 and 8 weeks after feeding a high-cholesterol diet (means ± SEM).

In the rabbits which were fed with high fat chow for 8 weeks, serum cholesterol levels increased consistently - no significant differences between the Revacept-dosed group and the control animals occurred (please see Figure 5). Mean body weights did not differ significantly between Revacept-treated animals and control animals during the investigation period.

### Acetylcholine-induced Vasoreactivity

**Figure 6:**
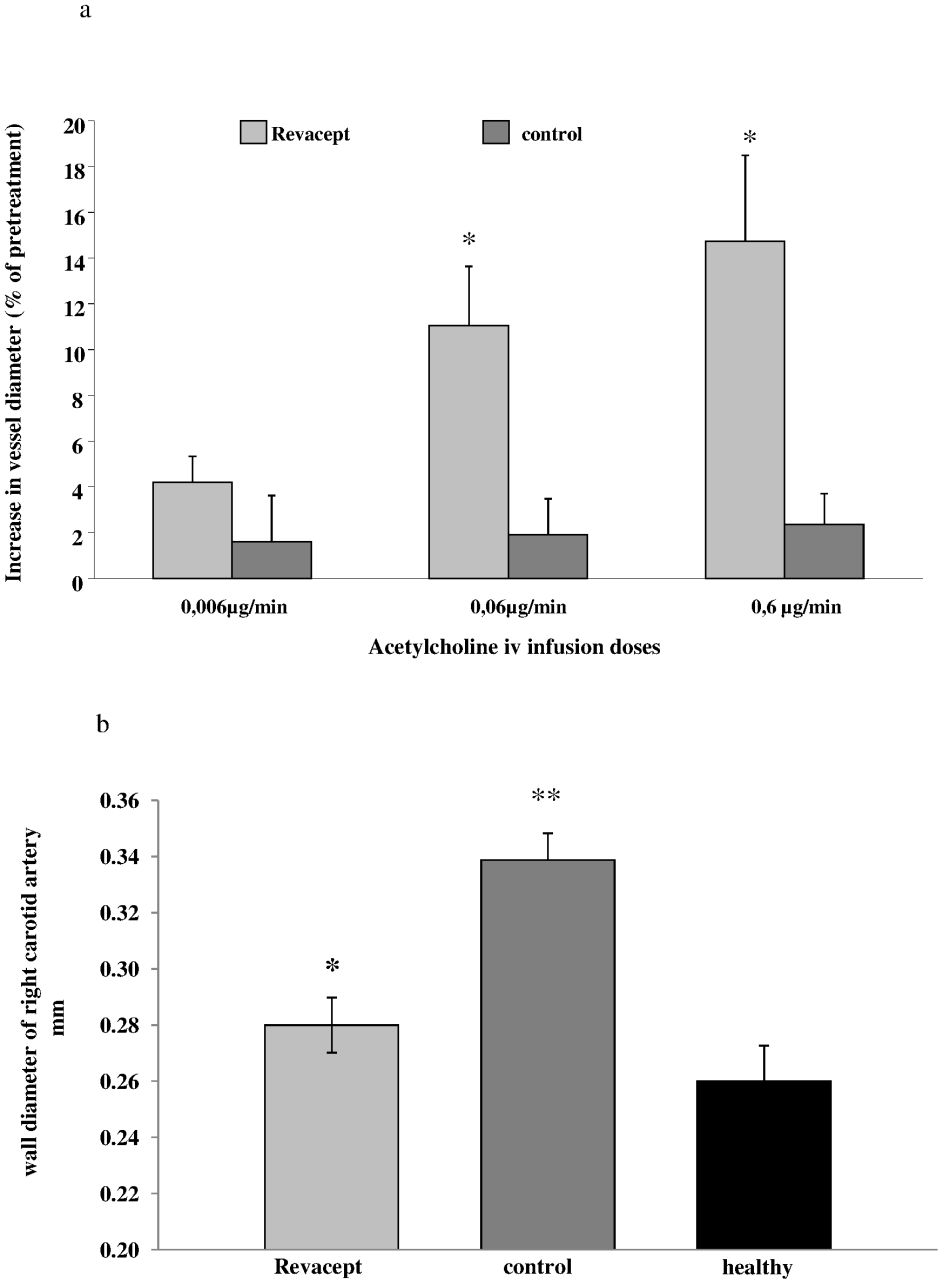
Endothelial dysfunction and arterial wall thickness was assessed in rabbits after 8 weeks of high cholesterol diet. (a) Vascular ultrasound of the right common artery: The bars show increases of luminal diameter of the right common carotid artery [%] compared to baseline after infusion of three doses of acetylcholine in the Revacept and control groups. (b) Wall thickness of the right common carotid artery [mm] in rabbits. The mean ± SEM of 8 animals are shown. * indicates significant difference of p<0.05 versus cholesterol-fed, atherosclerotic control rabbits, and ** p<0.01 versus healthy rabbits (ANOVA).

In the cholesterol-fed rabbits, induction of vasodilation was determined as a measure of endothelial function by ultrasound-detected change in vessel diameter at the end of the experiment. A significant improvement of endothelial function was measured in Revacept-treated hypercholesterolemic animals compared to control animals. Vasoreactivity was significantly improved during acetylcholine infusion of 0.06 µg/min and 0.6 µg/min in animals after 4 weeks of treatment with Revacept twice weekly, when compared to vehicle-treated control animals (see Figure 6a).

### Thickness of the Vessel Wall

Additionally, a significant reduction of the vascular wall thickness was observed in Revacept-treated hypercholesterolemic animals compared to vehicle-treated control atherosclerotic animals. Additionally, the cholesterol-fed Revacept group even showed no significant difference compared to healthy animals without treatment (see Figure 6b).

### Plaque Size Measurements in en Face Preparations (Macroscopic Evaluation)

**Figure 7:**
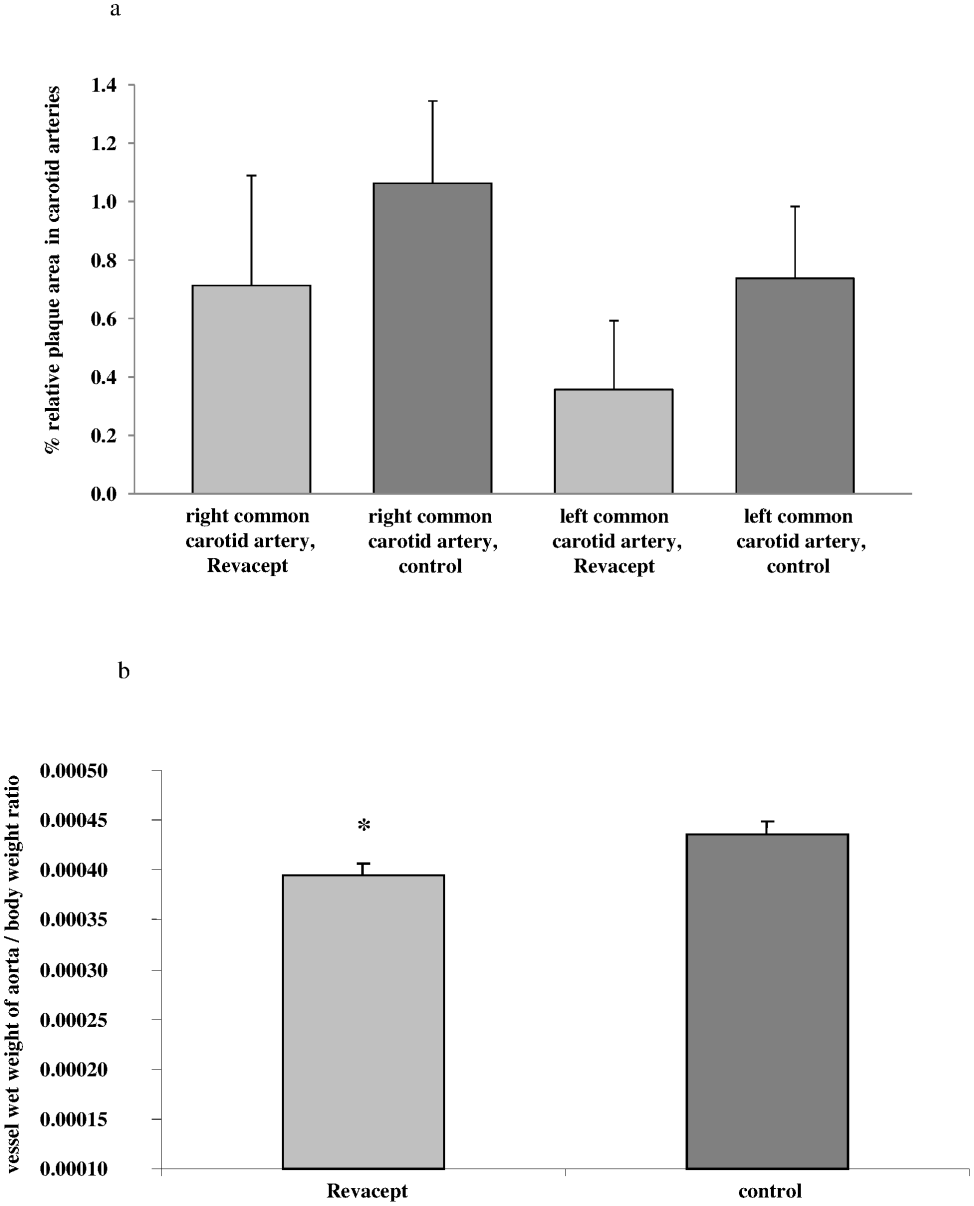
Macroscopic and histological assessment of atherosclerosis in cholesterol-fed rabbits. (a) Plaque size [%] was determined in macroscopic en face preparations after sudan red staining of the common carotid arteries. The relative lesion area was expressed as percentage of the total vessel area of the common carotid artery. (b) Vessel wet weight to body weight ratio was determined. The mean ± SEM of 8 animals are shown. * indicates significant difference of p<0.05 versus cholesterol-fed atherosclerotic control rabbits.

**Figure 8:**
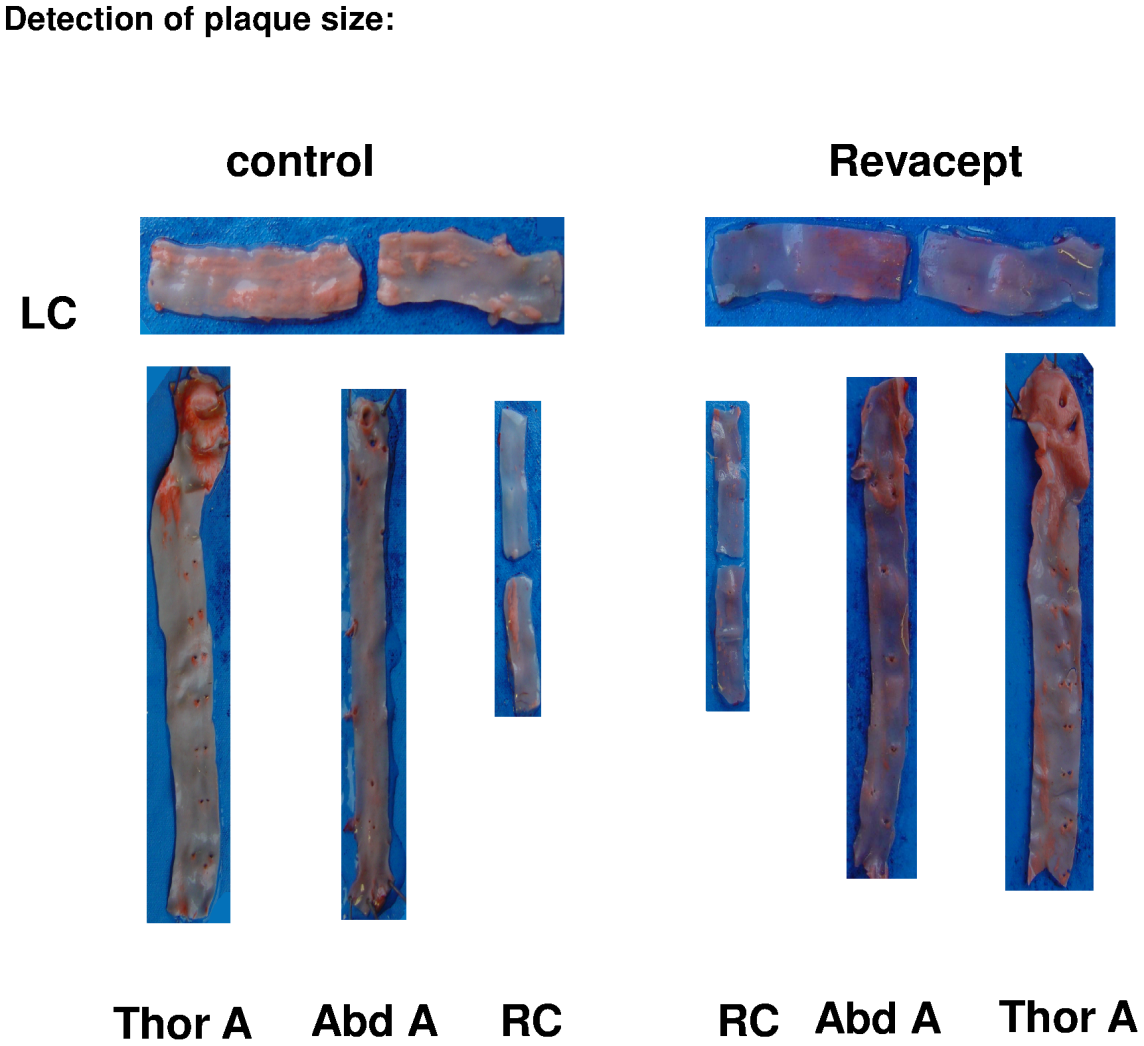
Representative images of plaque extensions in artery sections. LC: left carotid artery; RC: right carotid artery, Thor A: thoracic aorta, Abd A: abdominal aorta.

In Revacept-treated hypercholesterolemic animals, plaque size tended to be reduced in both common carotid arteries and in the abdominal aorta, although these differences did not reach statistical significance (see Figure 7a). Representative images are shown in Figure 8. Additionally, Revacept-treated hypercholesterolemic rabbits showed a significant reduction of the vessel weights of their aortae compared to controls (see Figure 7b).

### Immunostaining for Determination of the Density of Macrophages and Lymphatic Cells in the Vessel Wall Intima

**Figure 9:**
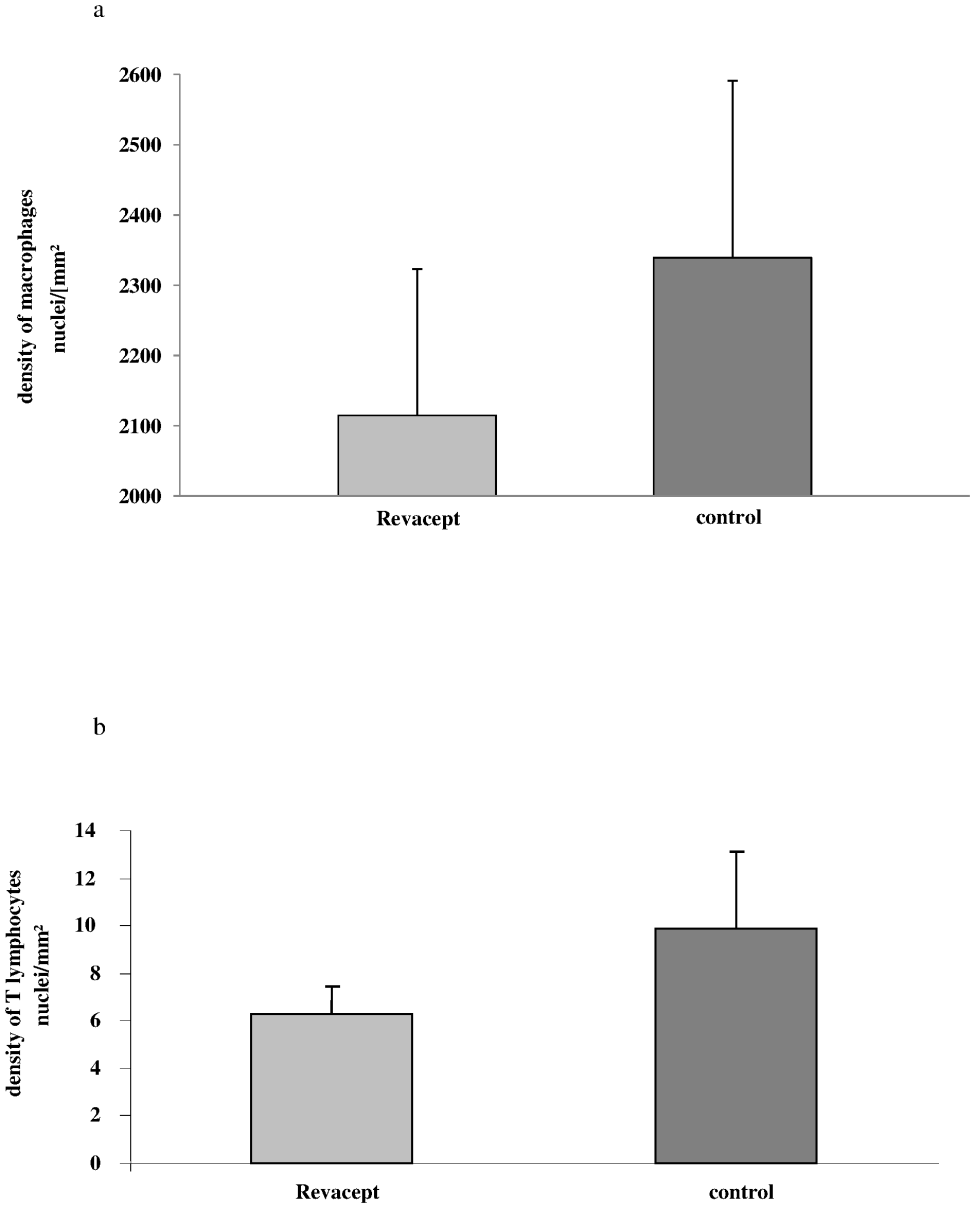
The inflammation in the arterial wall was assessed by immune-histology in carotid artery sections of cholesterol fed rabbits. (a) The density of macrophages was determined using specific anti-RAM antibodies.(b) The density of T-lymphatic cells was determined with specific anti CD 4 antibodies. The mean ± SEM of 8 animals are shown.

**Figure 10:**
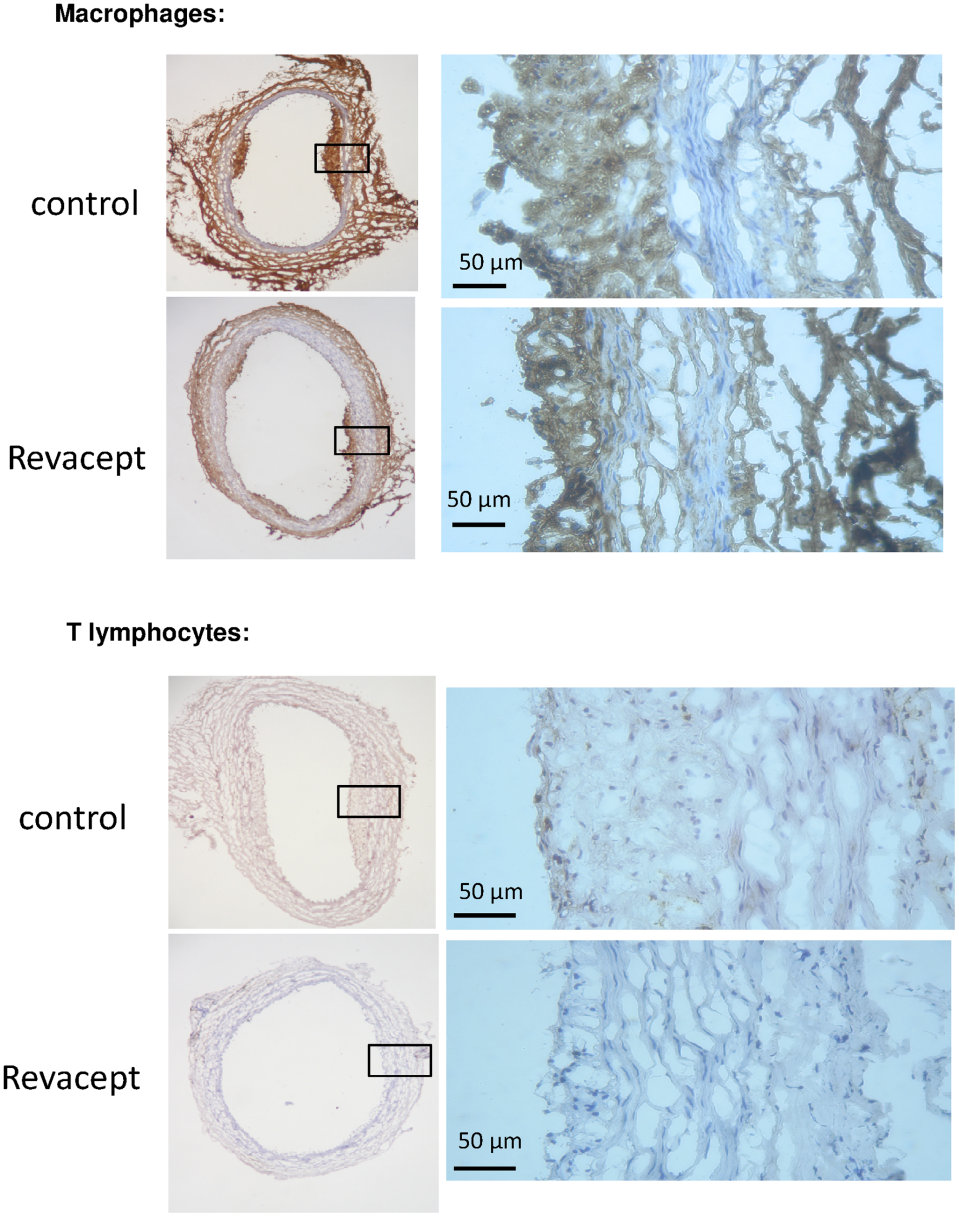
Representative immuno-histological images of macrophage and T lymphocyte stains of carotid artery sections.

The density of macrophages and lymphatic T-cells was statistically not different from controls - it tended to be reduced in the intima of the brachiocephalic trunk in Revacept-treated hypercholesterolemic animals compared to controls (see Figures 9a and 9b). Representative immuno-histological images are shown in Figure 10.

### Bleeding Times in Rabbits

30 minutes after intravenous administration of 2 mg/kg Revacept, bleeding time was 173±61 seconds, whereas it was 161±56 seconds after IV administration of vehicle only (n = 8 animals for each condition). There was no significant difference between the groups.

### Measurement of Bleeding Time in Mice

Bleeding times were also measured in mice by a standardized tail clipping protocol. These extended measurements could not be all done in rabbits, since it would have meant too many experiments in that species.

**Figure 11:**
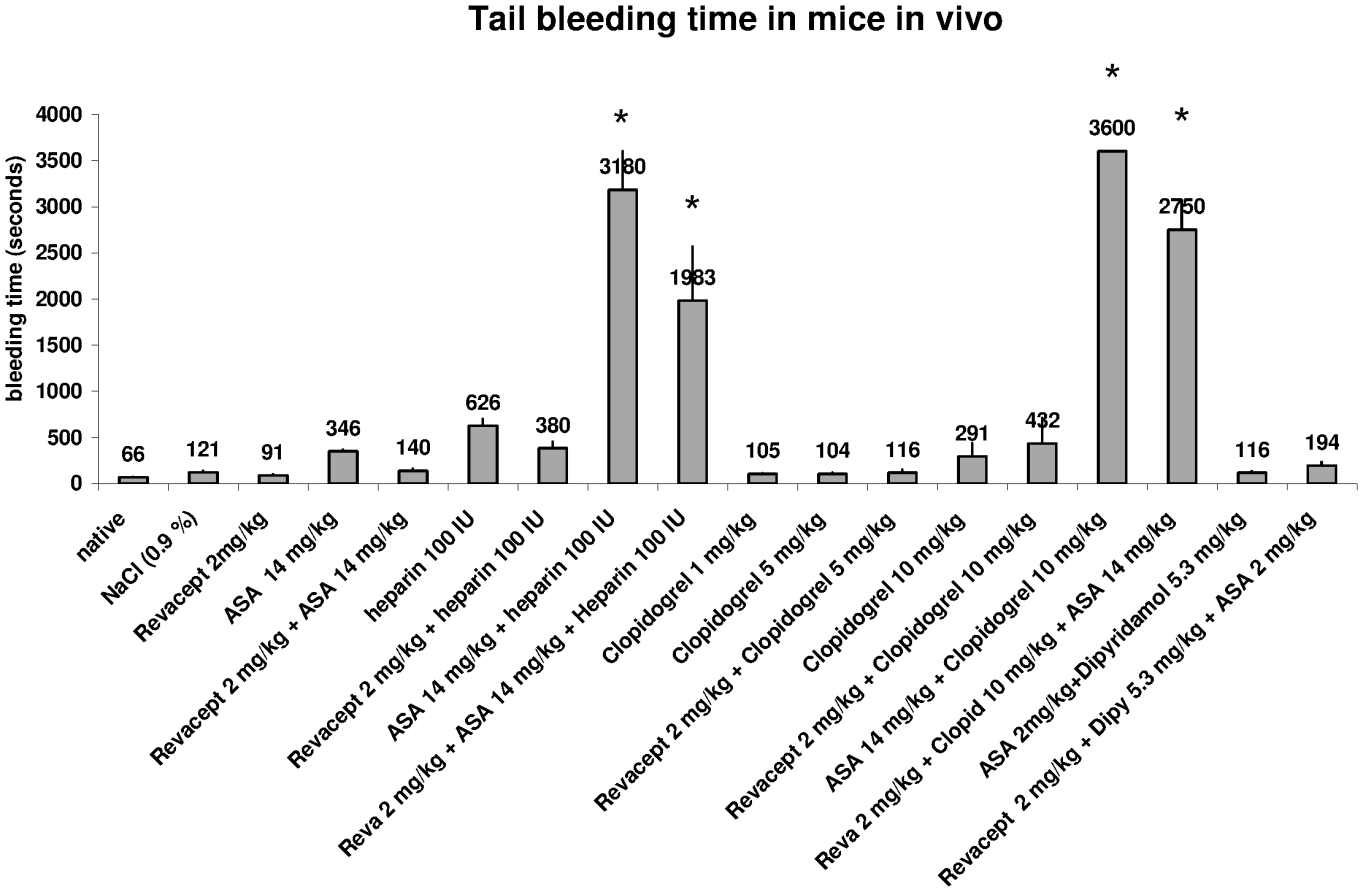
Tail bleeding time was assessed after tail clipping in mice. Revacept was investigated alone and in combination with existing drugs. The means ± SEM of n = 8 animals per group are demonstrated. Stars (*) indicate significant differences, p<0.05 versus native, untreated mice, or vs. mice which were only treated with 2 mg/kg Revacept (ANOVA).

A marked prolongation of bleeding time was induced after administration of established drugs such as Aspirin (14 mg/kg body weight IV), Heparin (100 IU IV), or clopidogrel (1 mg/kg body weight; 5 mg/kg body weight or 10 mg/kg body weight p.o) in mice. These doses are equivalent to standard or emergency doses with respect to per kg bodyweight doses used in patients with acute coronary syndromes. The doses correspond to 1000 mg Aspirin, 600 mg Clopidogrel, or 7500 IU Heparin per patient. Combining these drugs with Revacept (2 mg/kg) which corresponds to a dose of 160 mg per patient had no additional effect on bleeding times (see Figure 11).

## Discussion

In this study, we investigated the effects of an inhibition of GPVI-mediated platelet activation by Revacept on acute thrombus formation and on the progression of atherosclerosis in rabbits. Moreover, we intended to characterise the safety of Revacept with specific focus on bleeding complications in combination with other widely used anti-thrombotic drugs. Revacept dose-dependently reduced thrombus formation after endothelial injury. A significant improvement of endothelial dysfunction and vessel wall thickness was detected by in vivo vascular ultrasound after 4 weeks of Revacept in rabbits on high-fat diet. Finally, Revacept did not worsen the bleeding time in mice when applied in combination with widely used anti-thrombotic drugs in relevant doses.

It was demonstrated by various investigations that a high percentage of patients with acute coronary syndromes (ACS) had multiple ruptured plaques that induced atherothrombosis. Moreover, endothelial dysfunction was strongly associated with inflammation during advanced platelet aggregation and thrombus formation [12]. An increase in endothelial production of pro-coagulatory agents results in a thrombogenic vascular environment leading to thrombus formation by ruptured or erosive plaques [13]. It was a major finding of this study that treatment of atherosclerotic rabbits with Revacept also had a profound effect on the acetylcholine-induced endothelial function as determined by vascular ultrasound. Earlier studies had shown that Revacept prevents platelet adhesion [1] and this leads to a decrease of the inflammatory response in the endothelium [13]. Thus, the improvement of the endothelial function by Revacept is most likely due to a direct anti-platelet effect and subsequent to reduction of the inflammatory response by activated platelets. In earlier studies of our group, we showed that atheroprogression in two different atherosclerosis models was inhibited in the presence of Revacept [8], [14]. In these studies, both repeated administration of recombinant Revacept in ApoE −/− mice and gene transfer of GPVI-Fc to the carotid artery in cholesterol-fed rabbits were performed. Both interventions resulted in a notable decrease of atheroprogression in the arteries of either ApoE −/− mice [14] or cholesterol-fed rabbits with a significant reduction of plaque extension [8]. However, the expression of GPVI-Fc caused by an attenuated adenovirus is rather unfavorable due to induction of inflammation per se.

In vivo administration of a monoclonal anti-GPVI-antibody (JAQ1) in ApoE (−/−) mice after vascular injury reduced aggregation and adhesion of platelets at the arterial vessel wall [2]. Moreover, chronic administration of JAQ1 also markedly reduced atheroprogression in ApoE −/− mice [14]. Thus, GPVI seems to be a necessary regulator of platelet activation both in acute and chronic vascular syndromes. On the other hand, treatment with anti-GPVI-antibodies caused complete internalization and depletion of GPVI on platelets [2]. Thereby, these anti-GPVI antibodies, such as JAQ1, induced a strong and transient thrombocytopenia and significantly prolonged increase of the bleeding time [15]. In a controlled phase I study, we recently demonstrated in healthy human volunteers that Revacept had no pro-arrhythmic effect. Systolic blood pressure values, heart rate and ECG were all within the normal range after administration of Revacept. There was also no influence on the bleeding time, nor influence on the general haemostasis as assessed by partial thromboplastin time or INR in the subjects [10]. In this study we additionally demonstrate that Revacept did not prolong bleeding time after tail clipping in mice even when applied in combination with the most common used anti-thrombotic drugs. In contrast, the use of of 1000 mg aspirin or 600 mg clopidogrel caused excessive prolongation of bleeding time. The combination with Revacept with either drug, however, had no additional effect.

Concerning the data obtained with clopidogrel, one publication showed prolongation of bleeding time after double dosing of 3 mg/kg bw clopidogrel over 2 days in anaesthesized mice [16]. In our study, single dosings were compared, so that a lack of prolongation after 5 mg/kg seems plausible. Accordingly, thigh bleeding time was not altered after single dosing of 5 mg/kg clopidogrel in pigs, and moderately increased after 10 mg/kg [17].

Thus Revacept seems to possess unique features as an effective anti-platelet drug without the inherent side effect of increased bleeding.

The reason for using rabbits was to show that Revacept is not only an effective drug in the treatment of atherosclerosis in mice [14]. The use of another animal model to demonstrate efficacy can avoid drawing premature conclusions for the further drug development. Particularly, it was shown that other anti-atherosclerotic drugs such as statins fail to show efficacy in ApoE−/− mice [18]. In this manuscript, we show that Revacept is effective in reducing the morphological changes and in particular the endothelial dysfunction in two independent in vivo atherosclerosis models [8]. In accordance with previous studies it prevents platelet thrombus formation at vascular lesions [1]. Thus Revacept seems to impact on the known ligands in the atherosclerotic plaque after plaque rupture (i.e. collagen) and further ligands present on the activated atherosclerotic endothelium which shows no obvious lesions (i.e. fibronectin [8]).

Limitations of the study: Mouse tail bleeding times were investigated for some measurements of drug combinations in the current study, because the use of such a relevant animal number in other animal species would not have been compatible with local Ethics Committeés regulations. Investigation of this parameter in mice cannot entirely predict bleeding in humans, although it is often used to obtain a first idea on the respective bleeding risk.

Revacept therefore promises to be a useful efficient drug to interrupt platelet-triggered short-term thrombosis, but also long-term endothelial dysfunction and plaque progression in atherosclerosis, and does not incur the risk of bleeding complications as conventional anti-platelets drugs do.
